# Unpacking EFL Teacher Self-Efficacy in Livestream Teaching in the Chinese Context

**DOI:** 10.3389/fpsyg.2021.717129

**Published:** 2021-08-05

**Authors:** Honggang Liu, Wenxiu Chu, Ye Wang

**Affiliations:** School of Foreign Languages, Northeast Normal University, Changchun, China

**Keywords:** teacher self-efficacy, senior high school teachers, livestream English teaching, instructional self-efficacy, technological self-efficacy

## Abstract

Teacher self-efficacy has long been researched in the context of face-to-face teaching, but it has received less attention with regard to online teaching. To address this gap, the current study utilized a questionnaire adapted from Lin and Zheng as the major instrument and supplementary interviews to examine teacher self-efficacy in livestream teaching in the Chinese context. Exploratory factor analysis results from 486 senior high school English as a foreign language (EFL) teachers in China yielded a two-factor structure of teacher self-efficacy comprising instructional self-efficacy and technological self-efficacy. Across the sample, EFL teachers had moderate-to-high self-efficacy in general, and they showed higher levels of technological self-efficacy than instructional self-efficacy. The interview data also indicated a fluctuation in technological self-efficacy at the onset of livestream teaching compared to 1 month into livestream teaching. This study results offer some useful suggestions for enhancing teacher self-efficacy.

## Introduction

Self-efficacy, that is, self-perception of individuals of their competence in executing specific tasks (Bandura, 1977), has attracted considerable attention in teacher education (Tschannen-Moran and Woolfolk Hoy, 2001; Gilbert, 2005; Choi and Lee, 2016; Hoang and Wyatt, 2021). Teacher self-efficacy is conceptualized as a teacher's judgment of his/her own competence in managing the classroom, engaging students, and performing assigned teaching tasks (Tschannen-Moran et al., 1998). As English as a foreign language (EFL) is both domain specific and task specific (Bandura, 1997; Tschannen-Moran and Woolfolk Hoy, 2001), we were inspired to explore its teacher self-efficacy.

Most prior studies on EFL teacher self-efficacy were carried out in the context of traditional face-to-face classroom teaching, whereas EFL online teaching has received scant attention. The COVID-19 pandemic has caused significant damage to the lives of people, but it has also accelerated the development of online English language education (Carrillo and Flores, 2020; Kumar et al., 2020) and drawn worldwide attention to online English teaching modes (Gao and Zhang, 2020; Pu, 2020). To curb the spread of COVID-19, different countries enacted policies to enforce the replacement of traditional classroom learning with online learning (Carrillo and Flores, 2020). For example, the Ministry of Education in China called for a nationwide move to online teaching to ensure that while classes were suspended, learning would continue (Ministry of Education of the People's Republic of China, 2020).

Facing the sudden and unexpected challenges of the online environment, teachers in all disciplines (including language education) endeavored to learn how to design and engage in online teaching and make the necessary psychological adjustments. Against this backdrop, some researchers have studied language teacher psychology in the online teaching environment, for example, in relation to cognition (Gao and Zhang, 2020), work buoyancy (Anderson et al., 2021), and anxiety (Li et al., 2020). However, examinations of teacher self-efficacy in online English teaching are scarce. An in-depth understanding of this area will allow teacher educators or school administrators to improve the self-confidence and engagement of teachers at a pedagogical level and look for patterns in language teacher efficiency at a theoretical level. To address this aim, the current study utilized a questionnaire-based quantitative approach with supplementary interviews to explore patterns and levels of EFL teacher self-efficacy in online teaching in mainland China.

## Literature Review

### Defining EFL Teacher Self-Efficacy

Teacher self-efficacy may be generally defined as the belief of teachers in their competence to enhance the academic outcomes of students, engage students in the classroom, successfully carry out teaching tasks, and achieve teaching goals (Bandura, 1986; Campbell, 1996; Tschannen-Moran and Woolfolk Hoy, 2001; Gilbert, 2005; Hoang and Wyatt, 2021). This definition implies that teacher self-efficacy has a multidimensional structure. Akbari and Tavassoli (2011) proposed that EFL teacher efficacy is a multifaceted construct, including efficacy in teaching language skills, efficacy in teaching language components, efficacy in error correction, and assessment efficacy. Chan et al. (2010) examined language teacher efficacy in reading, listening, speaking, and writing and described self-efficacy on the basis of four language skills. Since self-efficacy is both task specific and context specific in nature (Wyatt, 2018), a shift in context to online teaching involves the confidence of teachers in their ability to teach online (Corry and Stella, 2018). In the English livestream teaching environment, we defined teacher self-efficacy as a teacher's self-perception of his/her ability to impart English language knowledge, manage software to carry out effective online English teaching, and strategically engage students in their online teaching.

### Studies on Language Teacher Self-Efficacy

For decades, a large body of research has been compiled that explores language teacher self-efficacy around three themes: internal and external factors influencing teacher self-efficacy, the correlation between teacher self-efficacy and other psychological factors, and the structure of teacher self-efficacy. Wang et al. (2004) research showcased significant treatment effects of the vicarious learning experience and goal setting on self-efficacy beliefs about technology integration. Moreover, Robinia and Anderson (2010) found a positive correlation between the highly scored self-efficacy of teachers over their online teaching and their mastery and preparation experience. Similarly, Horvitz et al. (2015) revealed internal factors of self-efficacy of teachers in their online teaching, including their perception of student learning, satisfaction with online teaching, and future interest in it.

Factors such as years of teaching experience (Campbell, 1996; Gilbert, 2005; Scherer et al., 2015; Shao, 2017; Hoang and Wyatt, 2021), language proficiency (Choi and Lee, 2016; Wyatt and Dikilitas, 2019), professional development programs (Karimi, 2011; Zonoubi et al., 2017; Lee and Davis, 2020), school environment (Shao, 2017), and practicums (Atay, 2007; Cabaroglu, 2014) have also been found to exert influences on teacher self-efficacy. In Kissau and Algozzine's (2015) study, EFL pre-service teachers became less confident and showed low self-efficacy in completing online teaching tasks. Scherer et al. (2015) found that EFL teacher self-efficacy had a significantly positive correlation with the utilization of information and communications technology (ICT) but a negative correlation with the age of teachers. Furthermore, Shao's (2017) research unveiled medium levels of EFL teacher self-efficacy in relation to teaching strategies and techniques, classroom organization and management, and efforts of teachers to develop affective attitudes and cultural awareness in students. Significantly, Yang's (2018) investigation of 150 EFL teachers' technological pedagogical content knowledge (TPACK) structure, referring to knowledge of the subject matter; modern technologies; and teaching strategies, methods, and procedures (Koehler and Mishra, 2005; Kormos and Nijakowska, 2017; Yang, 2018), revealed a positive relationship between TPACK and technology integration self-efficacy.

In fact, previous research has indicated positive and negative correlations between EFL teacher self-efficacy and other psychological factors, such as emotional intelligence (Kostić-Bobanović, 2020), teacher identity (Canrinus et al., 2012), occupational commitment (Gilbert et al., 2014; Huang et al., 2021), job satisfaction (Skaalvik and Skaalvik, 2014; Hampton et al., 2020; Safari et al., 2020; Huang et al., 2021), and teacher burnout (Skaalvik and Skaalvik, 2014). Schmidt et al.'s (2009) study uncovered positive correlations between teachers' technological self-efficacy and knowledge of technology, pedagogy, and content. Moreover, Skaalvik and Skaalvik (2014) found that English teachers' self-efficacy positively predicted their involvement and satisfaction with work and negatively predicted their emotional burnout. Similarly, studies by Safari et al. (2020) and Huang et al. (2021) showed positive correlations between teacher self-efficacy and job satisfaction. In addition, Kormos and Nijakowska (2017) discovered stronger self-efficacy among teachers after they completed a massive open online course (MOOC), where the more work they completed in the MOOC, the stronger their post-course self-efficacy beliefs became.

Regarding research on the structure of teacher self-efficacy, several scales or questionnaires have been developed and validated. The most frequently used instrument has been the Teacher Self-Efficacy Scale (Tschannen-Moran and Woolfolk Hoy, 2001). This scale has a long form (24 items) and a short form (12 items). Both have three dimensions—namely, efficacy in instructional strategies, efficacy in classroom management, and efficacy in student engagement. Subsequent researchers have adapted this scale to fit the English language teaching context (Chacón, 2005; Atay, 2007; Rastegar and Memarpour, 2009). As online teaching became more popular, researchers began to examine language teacher self-efficacy in the online context. Robinia and Anderson (2010) modified the aforementioned Teacher Self-Efficacy Scale (Tschannen-Moran and Woolfolk Hoy, 2001) to test online teaching efficacy and consequently identified four factors—namely, self-efficacy in online student engagement, online instructional strategies, online classroom management, and the use of computers. Among these four factors, self-efficacy in the use of computers was emphasized as an emerging concept indicating that self-perceptions of teachers regarding their use of the Internet or other computer-based applications should be examined (Horvitz et al., 2015). Lin and Zheng (2015) used a self-report questionnaire to investigate the effects of language teacher self-efficacy on their teaching practices in online courses, which included an asynchronous and self-adaptive part (courses in Spanish, French, German, and Japanese) and a synchronous session per week in a Chinese language course *via* audio conferencing. Their study examined both instructional and technological self-efficacy. The former emphasized the confidence of teachers in promoting language learning by students, including guiding them to finish assignments or homework and increasing their interest in learning. The latter focused on confidence of the teachers in using technological resources in language teaching and learning activities—that is, successfully teaching language content using appropriate technology and mentoring students to use technology appropriately. Furthermore, Siddiq et al. (2016) developed a scale to test teachers' emphasis on the development of students' digital information and communication skills (TEDDICS) in traditional language classroom teaching, and they identified a positive correlation between TEDDICS and self-efficacy in ICT.

The aforementioned studies shed light on a growing research trend of studying self-efficacy of teachers against the backdrop of greater concern over online teaching. Online teaching situates language teachers in a more complex context where teacher self-efficacy may be different compared to traditional face-to-face classroom teaching where technology-related efficacy is involved (e.g., Lin and Zheng, 2015). Clarifying the internal structure of teacher self-efficacy in online education can deepen our understanding of the correlation between self-efficacy and other psychological factors as well as the factors influencing teacher self-efficacy. Lin and Zheng (2015) based their scale on Bandura's self-efficacy theory (Bandura, 2006), but they did not validate the scale *via* EFA, which left a direction for the current study. In addition, research on online English teaching self-efficacy will contribute to improving online teaching effectiveness and enriching language teacher psychology research, which has long been neglected (Mercer and Kostoulas, 2018; Liu et al., 2020). To reach these aims, this study addressed the following questions:

1. What is the structure of EFL teacher self-efficacy in livestream teaching?
2. What are the levels of EFL teacher self-efficacy in livestream teaching?

## Methods

To mitigate the harmful influence of COVID-19 on education in China, the Ministry of Education published a document called “Classes suspended but learning continues.” Senior high school EFL teachers started teaching online in March 2020. Primarily, this took the form of livestream teaching *via* different online teaching platforms, like Ding Talk and Tencent. This study utilized a quantitative approach as the main method to explore the structure of online teaching self-efficacy and examine EFL teacher levels of instructional and technological self-efficacy. Interviews were used to triangulate the quantitative findings and yield further evidence to discuss.

### Participants

The sample comprised 486 senior high school EFL teachers from 18 provinces (359/74%), three autonomous regions (Inner Mongolia, Xinjiang, and Guangxi; 48/10%), and four municipalities directly under the central government (Beijing, Shanghai, Tianjin, and Chongqing; 79/16%). The sample comprised 429 (88.3%) female participants and 57 (11.7%) male participants, and 265 (54.5%) participants had no prior online teaching experience. In addition, eight teachers were selected for interviews on the basis of their provision of contact information (see Table 1). We researched senior high school EFL teachers due to the lack of research on self-efficacy in this group in either the online or traditional environment. The teachers had undergone almost no training in online teaching, which could pose challenges and provoke anxiety about their teaching. The findings of this study can serve as a reference for teacher educators, administrators, and policymakers to help secondary school teachers by alleviating negative emotions, boosting confidence, and indirectly improving online teaching efficiency (Akbari and Tavassoli, 2011; Ghonsooly and Ghanizadeh, 2013; Khani and Mirzaee, 2015). This research can also enrich studies on secondary school EFL teacher psychology.

**Table 1:** Demographic information of interviewees.

**Teacher**	**Gender**	**Age** **(years)**	**Educational** **degree**	**Years of** **teaching**	**TPACK**
Teacher 1	Male	32	Bachelor	8	NO
Teacher 2	Female	30	Bachelor	6	NO
Teacher 3	Male	25	Master	2	NO
Teacher 4	Female	31	Master	5	NO
Teacher 5	Female	29	Master	5	NO
Teacher 6	Female	28	Master	3	NO
Teacher 7	Female	30	Master	6	NO
Teacher 8	Female	25	Bachelor	2	NO

### Research Instruments

Two instruments were used in this study: a self-efficacy questionnaire and semi-structured interviews. The questionnaire aimed to collect the basic information of the participants and their levels of English online teaching self-efficacy. The semi-structured interviews were conducted to gather more in-depth information and explain the data collected in the questionnaires.

#### The EFL Teacher Self-Efficacy in Livestream Teaching Questionnaire

The EFL Teacher Self-Efficacy in Livestream Teaching Questionnaire (T-SE Q for short) was adapted from Lin and Zheng's (2015) Online Teacher Self-Efficacy Survey (see Appendix 1) and targeted EFL teachers and considered the features of EFL online teaching. We reworded the 13 items in the original survey due to the features of livestream teaching, since the online courses in Lin and Zheng's (2015) study were not all livestreamed. A five-point Likert scale ranging from one (strongly disagree) to five (strongly agree) was used to measure the degree of respondents' self-assessments on each item. Our T-SE Q obtained high reliability and validity (see Tables 2–4). Cronbach's alpha coefficients of each dimension of T-SE Q after EFA were above 0.70 (0.772–0.873), displaying high reliability.

**Table 2:** Internal consistency of the questionnaire (Lin and Zheng, 2015) and T-SE Q.

**Dimensions**	**Lin and Zheng (2015)**	**T-SE Q**
Instructional self-efficacy	0.690	0.895
Technological self-efficacy	0.920	0.772
Global teacher self-efficacy	Not mentioned	0.873

**Table 3:** Convergent validity of the T-SE Q.

**Factors**	**Items**	**Factor loadings**	**AVE**	**CR**
Instructional self-efficacy	1	0.699	0.716	0.882
	2	0.911		
	3	0.910		
Technological self-efficacy	4	0.377	0.469	0.767
	5	0.650		
	6	0.794		
	7	0.824		

**Table 4:** Pattern matrix of the T-SE Q.

**Items*I feel confident***	**Technological self-efficacy**	**Instructional self-efficacy**	**Communality**
An7: I can successfully teach relevant English language content using appropriate technology.	**0.824**	−0.063	0.760
An6: I can mentor students in appropriate uses of technology.	**0.794**	0.091	0.531
An5: I can help students when they have difficulty with the computer.	**0.650**	−0.023	0.445
An4: I can motivate my students to participate in the livestream class to support English learning.	**0.377**	−0.107	0.313
An3: I can motivate students to do their homework.	−0.010	**−0.911**	0.817
An1: I can keep students online on task on difficult assignments.	−0.037	**−0.910**	0.780
An2: I can motivate students who show low interest in livestream English learning.	0.135	**−0.699**	0.646
Cumulative % of variance explained	52.778	59.890	————

*Extraction method: principal axis factoring; two factors extracted. Ten iterations required*.

The validity of the questionnaire was measured by convergent validity and construct validity, the latter of which is reported in the first subsection of the section “Findings.” The whole scale had high convergent validity for loadings of each item, and all factors except technological self-efficacy were above 0.500 (Malhotra, 1993), while the average variance extracted (AVE) and composite reliability (CR) of the two factors were above 0.700 and 0.500, respectively (Hair et al., 2014).

#### Interview

A semi-structured interview was adopted as a supplementary tool for gathering more information about EFL teacher self-efficacy in online education. An interview protocol was employed to conduct the semi-structured interviews (see Appendix 3) to avoid straying from the topic while affording participants flexibility for reflection (Rose et al., 2020). It was designed in alignment with the research questions and focused on the two dimensions identified in the questionnaires—namely, instructional self-efficacy and technological self-efficacy.

### Research Procedure

Electronic questionnaires were distributed at the beginning of April 2020, in the middle of the semester of online teaching. Altogether, 486 valid questionnaires were collected by the end of May. The data were analyzed using SPSS 22.0. Two rounds of interviews were conducted in the first and fourth weeks of this livestream teaching. Each interview was done in Putonghua and lasted for ~45 min. All the interviews were audio-recorded with the permission of the participants and transcribed for further analysis after the discussions. All the transcriptions were checked by the interviewees and were then submitted to further analysis. The authors told the interviewees the purpose of the study and the principle of confidentiality of their identities and the recorded videos. They were also notified of their right to free withdrawal from the interview at any time if they felt any inconvenience or discomfort. As the interviewees had no contact with any of the three authors, there were no conflicts of interest between the authors and participants. All these procedures guaranteed the trustworthiness of this study.

## Findings

### Two-Dimensional Structure of EFL Teacher Self-Efficacy

The Kaiser–Meyer–Olkin measure of sampling adequacy was 0.865, suggesting that the questionnaire data were suitable for factor analysis. Additionally, Bartlett's test of sphericity yielded χ^2^ = 1760.532, *df* = 21, *p* = 0.000, indicating that the questionnaire data were suitable for factor analysis.

Thirteen items were analyzed using EFA with principal axis factoring and direct oblimin. The threshold of factor loading was set at |0.30|. Two factors were extracted and retained. As Table 4 shows, the cumulative percentage of total variance explained was 59.890% (above 55%, the referential line for EFA, cf. Plonsky and Gonulal, 2015), confirming that the two-factor structure was acceptable. Table 4 presents the two-factor structure of teacher self-efficacy. These two factors were named Technological self-efficacy and Instructional self-efficacy.

### Levels of EFL Teacher Self-Efficacy

With reference to the criteria for evaluating the levels of teacher self-efficacy (Choi and Lee, 2016), we set up the following standards to examine the degree of self-efficacy: low level (mean score lower than 3.0), moderate level (3.0–3.5), moderate-to-high level (3.5–4.0), and high level (higher than 4.0).

#### Moderate-to-High Levels of EFL Teacher Self-Efficacy in General

As Table 5 shows, teacher livestream self-efficacy stayed at a moderate-to-high level (*M* = 3.82, *SD* = 0.56). EFL teachers had a higher level of technological self-efficacy (*M* = 3.87, *SD* = 0.58) than instructional self-efficacy (*M* = 3.74, *SD* = 0.67).

**Table 5:** Descriptive analysis of the dimensions in T-SE Q.

**Dimensions**	**Max**	**Min**	**M**	**SD**
Technological self-efficacy	5.00	1.00	3.87	0.58
Instructional self-efficacy	5.00	1.00	3.74	0.67
Global self-efficacy	5.00	1.00	3.82	0.56

The interview data echoed the quantitative findings in Table 5:

Extract 1
*This was my first time conducting livestream teaching. Before entering the online classes, I received relevant training and became more confident in teaching students. However, sometimes, I was still worried about the effectiveness of my instructional strategies. (T3, 04-20-08)* ^1^
Extract 2
*Using technology to teach students is fresh to me. I often communicated with my colleagues about teaching problems in a WeChat group. They gave a lot of help using the software […] I had some teaching experience and practiced using it before class. I think it is more convenient and conducive to their English learning. (T5, 04-20-07)*
Extract 3
*Online classes are different from traditional face-to-face classes. We need to innovate our teaching strategies to attract students' attention. It is also necessary to communicate with my colleagues about teaching problems online. (T8, 04-20-08)*

As these three excerpts reveal, EFL teachers possessed confidence in teaching students after almost a month of online teaching due to colleagues' great encouragement and supportive training. Some teachers (e.g., T8) were also aware of the importance of innovating language teaching in online education, attracting the attention of students, and promoting their academic performance.

The instructional self-efficacy of EFL teachers is comparatively lower than their technological self-efficacy. In the second round of interviews, teachers explained as follows:

Extract 4
*I usually let students do some exercises after class. Their parents scanned or took photos of the answers and sent them to my email. I checked the e-versions of the assignments and then offered my feedback in the next class on some common problems. I also taught them some key vocabulary and grammar […] It was a little difficult to implement online task-based or communicative language teaching. Students were unwilling to speak English, and they liked typing simple words or pasting others' words […] I didn't know whether they had truly mastered the language knowledge. (T6, 04-20-06)*
Extract 5
*I didn't have enough time in class to achieve my teaching aims. However, lesson preparation time seemed to have increased, because I had to read teaching materials in advance and observe others' lessons […] The biggest challenge was that I couldn't see all of them. I didn't know if they listened to the class seriously. (T3, 04-20-08)*
Extract 6
*Class time was limited. It was inconvenient to interact with students in live online lectures, and students seemed to be unwilling to receive a video or voice call to answer my questions in class. (T2, 04-20-06)*

The interviews indicated that instructing students online presented difficulties for the EFL teachers. Specifically, they had long working hours but not enough class time to accomplish their teaching aims successfully. Constraints of the livestream teaching platform and students' unwillingness to speak online, in most cases, prevented teachers from effectively interacting with students and understanding their academic performance.

#### Moderate-to-High Levels of Teacher Technological Self-Efficacy

The data suggested that the EFL teachers had moderate-to-high levels of technological self-efficacy (*M* = 3.87, *SD* = 0.58). The four items under this dimension indicated high-to-moderate levels of confidence in teachers (see Table 6) in motivating students to participate in the livestream class (*M* = 4.31, *SD* = 0.60), mentoring students in technology use (*M* = 3.81, *SD* = 0.81), teaching language content using technology (*M* = 3.75, *SD* = 0.76), and helping students solve technological difficulties (*M* = 3.62, *SD* = 0.84).

**Table 6:** Descriptive analysis of technological self-efficacy scores.

**Items**	**Max**	**Min**	**M**	**SD**
An4: I feel confident that I can motivate my students to participate in the livestream class to support English learning.	5.00	1.00	4.31	0.60
An6: I feel confident that I can mentor students in appropriate use of technology.	5.00	1.00	3.81	0.81
An7: I feel confident that I can successfully teach relevant English language content using appropriate technology.	5.00	1.00	3.75	0.76
An5: I feel confident that I can help students when they have difficulty with the computer.	5.00	1.00	3.62	0.84

EFL teachers displayed higher technological self-efficacy in dealing with daily teaching tasks (An7), such as motivating students to participate in the livestream class (An4) and mentoring them in appropriate technology use (An6). However, the qualitative findings indicated that technological self-efficacy was low at the outset of online teaching and gradually increased with the development of technological skills in teachers. The following data from the first round of interviews illustrate this:

Extract 7
*I faced many difficulties. It was my first time conducting livestream teaching, so I worried about its effect. Schools already trained us about technology use, and we also practiced using the software. I think I can manage the online classroom. (T4, 03-20-04)*
Extract 8
*Many teachers had no experience of online teaching. We did not have enough time to prepare for this new form of teaching […]. However, the training provided by the school and the support from colleagues helped me finish teaching tasks. (T6, 03-20-05)*

The above interview data indicated that EFL teachers seemed not to be very confident in applying technology in online language teaching at the outset because they lacked rich online teaching experience and sufficient time to strengthen their technological skills in advance. However, due to the technological training they received and their cooperation with colleagues after online teaching began, their technological self-efficacy gradually increased.

#### Moderate-to-High Levels of Teacher Instructional Self-Efficacy

Instructional self-efficacy consisted of three items. An1 was related to confidence of teachers in engaging students in livestream English learning, and An2 and An3 both focused on their confidence in motivating students to complete tasks in online education. Table 7 shows that the EFL teachers had moderate-to-high levels of instructional self-efficacy. Specifically, they were confident in their capacity to motivate students (*M* = 3.77, *SD* = 0.76) and, more specifically, to motivate them to finish homework (*M* = 3.74, *SD* = 0.72) and difficult assignments (*M* = 3.72, *SD* = 0.73).

**Table 7:** Descriptive analysis of EFL teachers' instructional self-efficacy scores.

**Items**	**Max**	**Min**	**M**	**SD**
An2: I feel confident that I can motivate students who show low interest in livestream English learning.	5.00	1.00	3.77	0.76
An3: I feel confident that I can motivate students to do their homework.	5.00	1.00	3.74	0.72
An1: I feel confident that I can keep students online on task on difficult assignments.	5.00	1.00	3.72	0.73

The results from the second round of interviews also indicated that EFL teachers improved their instructional self-efficacy in the online context with the support of students and colleagues. Meanwhile, they focused more on student–teacher interaction in online language teaching and attempted to engage students in the classroom. This can be observed from the following extracts:

Extract 9
*At the beginning of online teaching, I just handed out many materials to students, but later, I realized it was difficult for them to understand immediately. Through communication with students and colleagues, I made some adjustments and prepared materials close to students' daily lives. (T4, 04-20-07)*
Extract 10
*We set up a WeChat group to seek solutions together and shared online teaching experiences. I also received a lot of encouragement from my colleagues […] Online teaching enabled me to learn more about my students. Students shared some exciting stories in class. I also told my English learning stories to motivate my students in their online learning at home. It seemed that our relationships got better. (T2, 04-20-06)*
Extract 11
*During online courses, I adopted several methods to attract students' attention and interact with the students. For example, I might suddenly call my students' names to check their attention and design some interesting activities. I think I can do it well. (T8, 04-20-08)*

The online teaching environment forced EFL teachers to adopt appropriate methods to interact with students and develop effective teaching strategies. Like T2, T4, and T8 emphasized, more interesting and suitable teaching materials needed to be prepared, such as sharing English learning experiences and telling life stories. Additionally, EFL teachers explored some simple but useful ways to manage their online classes. For example, T8 said, “I might suddenly call my students' names to check their attention.” On the whole, EFL teachers exhibited a relatively good level of instructional self-efficacy, believed in their abilities to successfully impart knowledge, and strategically engaged students in their online teaching.

## Discussion

### Structure of EFL Teacher Self-Efficacy

Factor 1 is named *instructional self-efficacy*, which focuses on EFL teachers' confidence in engaging students in relevant tasks and livestream teaching. It contains teachers' confidence in motivating students to complete an online task (An1) and homework (An3) and participate in livestream language learning (An2). This factor showcases confidence of teachers in their language teaching and in engaging students to some degree. It is closely associated with Bandura's (1986); Bandura (1997) and Lin and Zheng's (2015) notion of instructional self-efficacy and Tschannen-Moran and Woolfolk Hoy's (2001) notion of teacher self-efficacy. Based on the study by Bandura (1997), Tschannen-Moran and Woolfolk Hoy (2001) conducted a survey to examine teacher self-efficacy and emphasized student engagement as an important factor of teacher self-efficacy. These factors were mirrored in Factor 1 in this study.

Factor 2 is related to *technological self-efficacy*, which highlights EFL teachers' self-judgments about their technological skills. It includes motivating students to participate in the livestream class (An4), helping them use technologies to facilitate their online learning (An6) and solve technological difficulties (An5), and using technology to successfully teach (An7). These abilities or strategies for using technology enabled the livestream teaching to move smoothly and rewarded teachers with high self-efficacy (Wang et al., 2004; Lin and Zheng, 2015).

Due to unpreparedness of teachers for livestream teaching and the inadequacy of TPACK, the teachers were trained to manipulate the livestream platform only at the beginning of the online teaching. However, they were still anxious about using modern technology to teach English. Most of the interviewees complained about having to prioritize transmitting knowledge over designing and organizing various teaching activities. They had limited time to explore how to take advantage of the functions provided by the platforms to enrich the types of teaching activities. Most of them were only familiar with basic functions of the platforms, such as how to control the volume and share their screen. They did not know how to organize interactive activities online. Furthermore, they did not assign technology-based tasks to the students; homework was completed in the traditional pencil-and-paper way, then scanned or photographed by parents, and finally emailed to the teachers. The teachers marked the homework and provided feedback to the students individually through WeChat instead of using the grading function of the platform. Therefore, three items relating to Lin and Zheng's (2015) notion of technological self-efficacy did not enter Factor 1 in this current study. The lack of concern about organizing collaborative activities in livestream teaching may be why item 5 (“I feel confident that I can encourage students to collaborate in practicing the English language online”) in Lin and Zheng's (2015) study was not retained in Factor 2 in the present study. Most teachers favored the traditional grammar translation method because it saved time, especially when there was uncertainty about internet speed and stability and anxiety over the potential of not delivering all the planned content. Thus, English was seldom spoken and used in the livestreamed classes, and this resulted in minimal exposure to English, which might have led to the removal of items 1 and 3 in Lin and Zheng's (2015) study.

### Levels of EFL Teacher Self-Efficacy

#### On the General Levels of EFL Teacher Self-Efficacy

In general, EFL teachers displayed moderate-to-high levels of self-efficacy in livestream teaching. This result shows the positive self-perceptions of teachers' own ability to execute online English teaching tasks and improve the academic performance of students. This finding is partially supported by what has been examined in teacher self-efficacy in traditional face-to-face classes (e.g., Shao, 2017; Kostić-Bobanović, 2020). Shao (2017) found that EFL teachers in China had a moderate-to-high level of self-efficacy, and Kostić-Bobanović (2020) argued that experienced EFL teachers showed a moderate-to-high level of self-efficacy.

One possible reason for this moderate-to-high level of teacher self-efficacy is teachers' previous mastery experience. Mastery experience has been regarded as the primary source of teacher self-efficacy (Bandura, 1997). A prior successful experience is likely to help teachers maintain confidence in their teaching ability (Robinia and Anderson, 2010; Chu et al., 2021) in the online environment. The interview data (i.e., T4 and T5) also revealed that EFL teachers faced many online teaching challenges but remained confident in online education, partly because of their teaching experience and repetitive online teaching practice.

Additionally, EFL teachers have shown high self-efficacy partly due to their strong professional identity (Canrinus et al., 2012) and occupational commitment (Gilbert et al., 2014). Interview data revealed that some EFL teachers (T1 and T7) with a stronger sense of self-efficacy viewed teaching as an attractive and meaningful profession and acknowledged the vital role of teaching in the academic and mental development of students. Self-efficacious teachers display higher levels of job satisfaction (Skaalvik and Skaalvik, 2014; Safari et al., 2020; Huang et al., 2021) and occupational commitment (Gilbert et al., 2014; Huang et al., 2021). Most teachers in this study also thought that teaching significantly influences children's growth, especially during the period of COVID-19. They believed they could provide aid in academic performance of students and daily lives to some degree through more teacher–student interaction. This positive self-evaluation of their teaching indicates that the teachers held a positive professional identity and self-efficacy.

Apart from internal factors, external factors, such as support resources provided by the schools and harmonious relationships between teachers, students, and other important persons, can explain this moderate-to-high level of teacher self-efficacy in livestream teaching. When the EFL teachers first began the livestream teaching, they were anxious due to their lack of similar experience. In response to this challenge, on the one hand, the schools or local teacher education institute organized online lectures on how to do livestream teaching, which offered scaffolding to teachers and bolstered their confidence (Karimi, 2011; Lee and Davis, 2020) that they would do well in livestream teaching. On the other hand, some teachers (T2 and T5) established WeChat groups on their mobile phones to share their online teaching experience and seek help from each other. This agentic engagement (Reeve and Tseng, 2011) facilitated their interaction with students by alleviating their anxieties and enhanced their self-efficacy (Gilbert et al., 2014). In the interviews, teachers (T2 and T8) reported their happiness in sharing daily stories and English learning experiences with students in their livestream teaching. This provided an effective lubricant to move the teaching forward. Teachers also received positive feedback on their teaching through verbal encouragement or emojis (e.g., 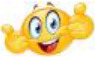) and built increasingly friendly relations with students. These harmonious interpersonal relationships may have assisted in strengthening teacher self-efficacy (Phan and Locke, 2015).

#### On the Levels of Teacher Technological Self-Efficacy and Instructional Self-Efficacy

Results of the descriptive analysis of teacher technological self-efficacy and instructional self-efficacy indicated moderate-to-high levels of both. Some favorable resources paved the path for EFL teachers to develop technological skills and knowledge, such as training provided by the schools, communication with colleagues, and repetitive practice by teachers in using the technology. The platforms were not difficult to operate. Therefore, the results indicate moderate-to-high levels of technological self-efficacy of teachers.

EFL teacher instructional self-efficacy was moderately high, which can be interpreted as follows. Teachers were fully devoted to the teaching preparations, including learning skills to manipulate the platforms, analyzing the teaching materials, and producing high-quality assignments. They also worked out every means of motivating students' engagement, such as sharing English learning stories (e.g., T2).

It is worth noting that EFL teacher technological self-efficacy is a dynamic developmental process. According to the quantitative data, 54.5% of participants had no online teaching experience and 67.5% did not receive sufficient online teaching training. These two numbers perhaps implied lower teacher self-efficacy at the outset of livestream teaching. From the interviews, we observed that some teachers (T3, T4, and T5) claimed that they were not confident in their livestream teaching initially. Their livestream teaching self-efficacy increased as the schools began to offer training on online teaching. More EFL teachers were familiar with utilizing technology to conduct online language teaching smoothly and gradually strengthen their technological self-efficacy and skills (Horvitz et al., 2015; Scherer et al., 2015; Kormos and Nijakowska, 2017). Training plays an important role in introducing livestream teaching knowledge to teachers (Karimi, 2011; Lee and Davis, 2020). Another possible explanation for low initial technological self-efficacy of EFL teachers is that they lacked TPACK. Schmidt et al. (2009) found positive correlations between technological self-efficacy of teachers and their TPACK. Successful experiences of traditional face-to-face teaching are not equal to those of successful online teaching practice (Cavanaugh et al., 2004; Kissau and Algozzine, 2015), which requires EFL teachers to integrate technical and pedagogical knowledge into their English teaching knowledge and content knowledge. Therefore, inadequate TPACK might have frustrated teachers when they felt incapable of meeting the challenges of manipulating a livestream platform, transforming teaching materials into digital versions, and interacting effectively with students. Fortunately, they took part in training on livestream teaching and enjoyed mutual support from colleagues, which increased their sense of self-efficacy after 1 month of livestream teaching.

The current research also uncovered that EFL teachers were more confident in tackling technological problems and comparatively less confident in effectively teaching students and managing classes in the online environment, supported by Shao (2017) and Kostić-Bobanović (2020). In addition, Robinia and Anderson (2010) and Hampton et al. (2020) found that educators had the most confidence in their computer skills and the least confidence in student engagement. Teacher self-efficacy is pertinent to student engagement, classroom management, and instruction strategies (Tschannen-Moran and Woolfolk Hoy, 2001; Robinia and Anderson, 2010). Livestream teaching demands that teachers balance platform manipulation and instruction, and interviewees reported that this caused them anxiety. Short training can aid teachers in handling livestream platforms, but it cannot address how to design and organize livestream teaching activities if the teachers lack TPACK. In the livestream classes, students provided feedback by typing simple words, since they were focused on a PowerPoint or other materials on the screen, and they had almost no time to provide prompt, detailed feedback to the teachers. It was difficult for the teachers to apply communicative language teaching (CLT) or task-based language teaching (TBLT) in their livestream teaching owing to the unstable internet and limitations of the platforms. Authentic activities in traditional face-to-face instruction, such as role play, are not easy to transfer to a livestream platform.

Furthermore, interview data indicated that some students seemed to avoid effectively interacting with their EFL teacher in the online environment, making teachers unclear about their teaching quality and easily affecting their instructional self-efficacy. Although online education offers teachers greater flexibility and autonomy compared to traditional classroom teaching (Corry and Stella, 2018), they must devote more time to lesson preparation to make the transition from offline to online education smooth. Numerous interviewees showed more confidence and willingness to teach in the offline environment.

## Conclusions

The current research explored the self-efficacy of EFL teachers in livestream teaching. It validated Lin and Zheng's (2015) online teaching self-efficacy questionnaire *via* EFA. The study contextualized the two-factor structure of teacher self-efficacy—namely, instructional self-efficacy and technological self-efficacy—in the high school livestream English teaching context. The interview data supported the quantitative findings and uncovered a fluctuation in technological self-efficacy from the lower level on the commencement of livestream teaching to a higher level after 1 month of instruction. These findings enrich studies on teacher self-efficacy, especially teacher self-efficacy in the online context, which is under-explored in language teacher education. The two-factor teacher self-efficacy model offers an example for future research to follow and revalidate.

The study has the following pedagogical implications. The results suggest that local teacher education institutions or high school authorities should provide EFL teachers with professional development programs and training on designing online teaching. According to this research, most EFL teachers are motivated to conduct online teaching before they possess the requisite skills and confidence, leading to poor job performance. Several studies have indicated that professional development programs and training are conducive to strengthening teacher self-efficacy (Murday et al., 2008; Karimi, 2011; Zonoubi et al., 2017; Lee and Davis, 2020). In this training process, they are able to learn up-to-date teaching methods and share their online teaching challenges and feasible problem-solving strategies with colleagues. Meanwhile, they may receive effective feedback or encouragement from others and foster a sense of collective self-efficacy (Gilbert et al., 2014), which are essential sources of teacher self-efficacy. In addition, it is necessary to set up a formative evaluation mechanism that contributes to building a positive professional identity. Effective feedback from authorities has a positive influence on teachers' identity. A fair and motivating evaluation enables teachers to establish a positive professional identity and commitment, thus improving teacher self-efficacy (Moslemi and Habibi, 2019; Chen et al., 2020) and strengthening the motivation to teach, especially in situations of adversity.

This study had some limitations that suggest directions for future research. Although the participants were all EFL teachers from different regions in China, both the interview and questionnaire sample sizes must be enlarged. The adapted online EFL teacher self-efficacy scale was a preliminary exploration. It is recommended that future studies broaden both the sample and research scope and validate the questionnaire used in this study to enrich the findings of teacher online self-efficacy research. Future researchers can also conduct longitudinal studies to explore how self-efficacy of teachers fluctuates and investigate how their self-efficacy relates to psychological variables (e.g., identity, well-being, emotional intelligence) in the online context.

## Data Availability Statement

The original contributions presented in the study are included in the article/Supplementary Material, further inquiries can be directed to the corresponding author.

## Author Contributions

HL conceptualization, data analysis, revision, supervision, and funding. WC and YW data analysis, draft, and revision. All authors contributed to the article and approved the submitted version.

## Conflict of Interest

The authors declare that the research was conducted in the absence of any commercial or financial relationships that could be construed as a potential conflict of interest.

## Publisher's Note

All claims expressed in this article are solely those of the authors and do not necessarily represent those of their affiliated organizations, or those of the publisher, the editors and the reviewers. Any product that may be evaluated in this article, or claim that may be made by its manufacturer, is not guaranteed or endorsed by the publisher.

## Footnotes

^1^In (T3, 04-20-08), T3 refers to the third interviewee and 20-04-08 represents the interview date, April 8, 2020.
