# Supplementary material for: Unpacking EFL Teacher Self-Efficacy in Livestream Teaching in the Chinese Context
Source: Front Psychol. 2021 Aug 5;12:717129. doi: 10.3389/fpsyg.2021.717129 (PMC8374736; doi:10.3389/fpsyg.2021.717129)
Supplement: Supplementary file 1 [file Table_1.pdf]

Appendix 1 The English Teacher Self-Efficacy in Livestream Teaching Questionnaire (Full Version)

Subscales	Items
Instructional self-efficacy	1. I feel confident that I can promote language acquisition even though there is no English target-language support available in the students' homes.
	2. I feel confident that I can keep students on online task on difficult assignments.
	3. I feel confident that I can increase students' retention of the English language.
	4. I feel confident that I can motivate students who show low interest in livestream English learning.
	5. I feel confident that I can encourage students to collaborate in practicing English language in online
	6. I feel confident that I can motivate students to do their homework.
Technological self-efficacy	7. I feel confident that I can successfully teach relevant English language content using appropriate technology.
	8. I feel confident that I can help students when they have difficulty with the computer.
	9. I feel confident that I can motivate my students to participate in livestream to support English learning.
	10. I feel confident that I can mentor students in appropriate uses of technology.
	11. I feel confident about assigning and grading technology-based tasks.
	12. I feel confident about using technology resources (such as spreadsheets, electronic portfolios, etc.) to collect and analyse data from student English performance scores to improve instructional practices.
	13. I feel confident that I can develop creative ways to cope with the constraints of the learning management system and continue to teach English effectively with technology.

(Adapted from Lin and Zheng, 2015)

Appendix 2 The English Teacher Self-Efficacy in Livestream Teaching Questionnaire (Final Version after EFA)

Subscales	Items
Instructional self-efficacy	1. I feel confident that I can keep students on online task on difficult assignments.
	2. I feel confident that I can motivate students who show low interest in livestream English learning.
	3. I feel confident that I can motivate students to do their homework.
	4. I feel confident that I can motivate my students to participate in livestream teaching to support English learning.
Technological self-efficacy	5. I feel confident that I can help students when they have difficulty with the computer.
	6. I feel confident that I can mentor students in appropriate uses of technology.
	7. I feel confident that I can successfully teach relevant English language content using appropriate technology.

Appendix 3 An Interview Protocol

1. Are there any teaching challenges that you face in the online context?
2. How do you deal with those challenges?
3. Are any changes in your feeling and teaching ability after a period of online teaching?
4. Are there any events or persons that give you a sense of achievement?
5. Are there any events or persons that make you depressed in online teaching?
